# Air in the Breast: A Rare Cause of Iatrogenic Pneumomastia

**DOI:** 10.7759/cureus.8447

**Published:** 2020-06-04

**Authors:** Shweta Paulraj, Prashanth Ashok Kumar, Navharsh Sekhon, Harvir Singh S Gambhir

**Affiliations:** 1 Internal Medicine, State University of New York (SUNY) Upstate Medical University, Syracuse, USA; 2 Internal Medicine, Adesh Institute of Medical Sciences and Research, Punjab, IND

**Keywords:** pneumomastia, breast, iatrogenic, peripheral line

## Abstract

Air in the breast tissue (pneumomastia) is a rare finding, more so without any history of procedures on the breast. We report the case of an 80-year-old lady who was found to have foci of gas in her right breast on a CT scan. On exclusion of other possible causes for the same, it was concluded that the air in the breast tissue was secondary to a peripheral intravenous line placement. To our knowledge, this is the first reported case of pneumomastia as a complication of peripheral intravenous line placement.

## Introduction

The breast tissue is composed of glandular and adipose elements held together by Cooper’s ligaments [1]. The normal radiographic appearance of the breast tissue on a CT scan can reveal varying densities, such as entirely fatty, scattered areas of fibroglandular density, heterogeneously dense and extremely dense as per the updated fifth version of the Breast Imaging Reporting and Data System (BI-RADS) glossary [2]. Air in the breast tissue is a rare finding on imaging and has been occasionally seen after procedures on the breast, such as breast augmentation [3]. Anaerobic infection such as gangrene of the breast is another cause of air in the breast and would commonly be associated with constitutional signs of infection [4]. Other cases of air in the breast have been seen from iatrogenic causes, such as mechanical ventilation and thoracostomy tube placement [5,6]. We report a rare case of air in the breast secondary to a peripheral intravenous line placement.

## Case presentation

**Figure 1:**
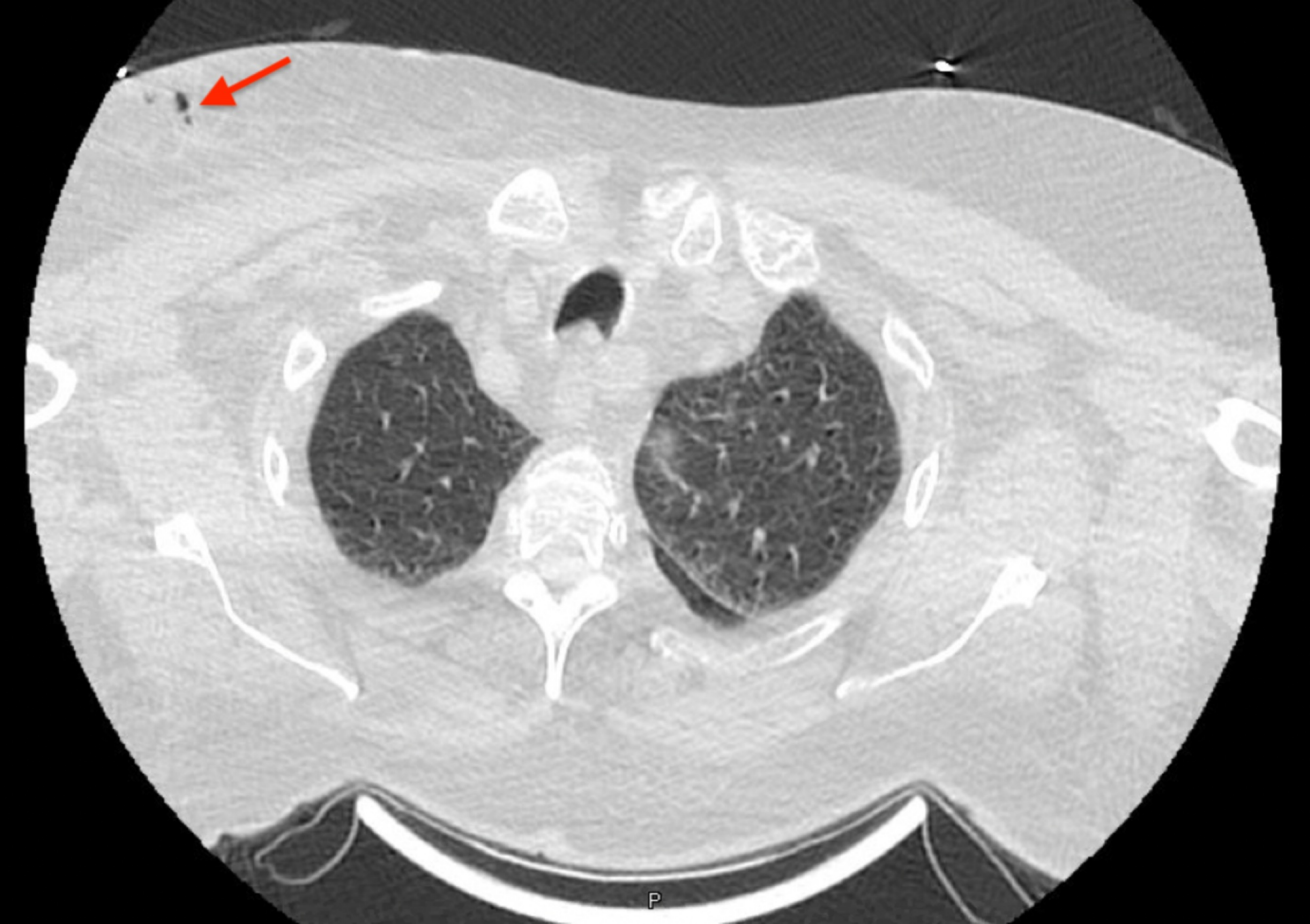
Air in the subcutaneous tissue of the breast (arrow)

An 80-year-old lady with a past medical history of hypertension, chronic obstructive pulmonary disease, atrial fibrillation, heart failure, chronic kidney disease, fibromyalgia, cerebrovascular disease and a non-active pituitary mass presented to the hospital with nausea, vomiting, fatigue and poor oral intake. Further workup revealed that she had panhypopituitarism, which was being managed in the hospital. During her hospital stay, she became lethargic likely secondary to delirium with electrolyte abnormalities from her pituitary disorder and required bilevel positive airway pressure (BiPAP) support for respiration. Her respiratory status continued to deteriorate with acute respiratory failure, which prompted further workup with a CT scan of the thorax. CT thorax revealed a finding of edematous changes isolated within the right breast area with several gas foci just superficial and inferior to the right clavicle (Figure 1).

The patient did not have any pain or discharge from the right breast. She did not have any fevers or chills during the hospital stay. Physical examination did not reveal warmth, erythema, induration or crepitus over the breast. Labs showed normal leukocyte counts during this course. The finding of air in the breast was therefore not attributed to infection. She was thoroughly evaluated for other possible causes of air in the breast based on prior case reports. She did not have any procedures over the right chest wall during the hospitalization. She did not have a central line placement, was not intubated but was placed on BiPAP for respiratory failure. She did not have a history of breast cancer and no recent mammogram, but reported that her mammograms from several years prior did not show any abnormal findings. After a thorough chart review, it was found that there was a peripheral intravenous line placement on the day prior to the CT thorax over the right upper extremity. It was attributed to being the most likely source of the edema and gas foci. The surgery team was consulted to ensure no surgical intervention was necessary. No surgical intervention was warranted given her lack of infectious symptoms and they recommended a follow-up if she noticed any changes to her breast. The patient has not had repeat imaging done since then and has not followed up for any complaints with regard to her breast.

## Discussion

The breasts are located within the subcutaneous layer of the thoracic wall. The entire glandular tissue of the breast is surrounded by the subcutaneous layer except in the region of the papilla [7]. There are several causes of subcutaneous emphysema in general. In a review of causes of subcutaneous emphysema in the upper extremity, one commonly reported cause of noninfectious emphysema is the injection of air or inert gas. This could be secondary to the use of compressed air tools or even a mishap during blood donation [8]. A small laceration or puncture wound could serve as a one-way valve for airflow into the soft tissues [9,10].

Air in the breast tissue has reported localized procedures on the breast, such as insertion of breast implants, during which air is introduced into the breast with the infiltration of a local anesthetic agent [3]. Iatrogenic pneumomastia is seen with pneumocystography and pneumoductography [11,12]. Subcutaneous emphysema in the breast tissue can also be a manifestation of pneumomediastinum due to various causes, such as amyopathic dermatomyositis, chest trauma, airway or esophageal rupture, endobronchial or esophageal procedures, mechanical ventilation or thoracotomy. These can result in air leakage through various tissue planes into the breast, thus resulting in the development of mammary emphysema [13-15]. When local causes of pneumomastia are not found, the possibility of air dissecting from more distal sites should be considered. There have been cases of pneumomastia on mammogram after laparoscopic surgery [16].

In our case, multiple gas foci with edematous changes were seen in the right breast on a CT chest, which was eventually attributed to a peripheral intravenous line placement in the right upper extremity after extensive workup to exclude breast, lung or mediastinal pathologies.

## Conclusions

Air in the breast is a rare finding and can have multiple potential etiologies. To our knowledge, this is the first case reported of gas in the breast (pneumomastia) after an intravenous line insertion. Awareness of benign etiologies such as this one, more so when the patient is otherwise asymptomatic, can help avoid extensive workup.
